# Effect of gold nanoparticle incorporation into oil-swollen surfactant lamellar membranes

**DOI:** 10.1063/4.0000041

**Published:** 2020-12-15

**Authors:** Michihiro Nagao, Robert Bradbury, Siyam M. Ansar, Christopher L. Kitchens

**Affiliations:** 1 National Institute of Standards and Technology Center for Neutron Research, 100 Bureau Drive, Gaithersburg, Maryland 20899-6102, USA; 2 Center for Exploration of Energy and Matter, Department of Physics, Indiana University, Bloomington, Indiana 47408, USA; 3 Department of Physics and Astronomy, University of Delaware, Newark, Delaware 19716, USA; 4 Department of Chemical and Biomolecular Engineering, Clemson University, Clemson, South Carolina 29634, USA

## Abstract

An oil-swollen surfactant membrane is employed to measure the effects of incorporated hydrophobically functionalized gold nanoparticles (AuNPs) on the structure and dynamics of the membranes. While maintaining an average AuNP diameter of approximately 5 nm, the membrane thickness was varied from 5 nm to 7.5 nm by changing the amount of oil in the membrane. The membranes become softer as the proportion of oil is increased, while the thickness fluctuations become slower. We attribute this to an increased fluctuation wavelength. Incorporation of AuNPs in the membrane induces membrane thinning and softening. Oil molecules surround the nanoparticles in the membrane and help their relatively homogeneous distribution. AuNPs significantly alter the membrane's structure and dynamics through thinning of the membrane, increased compressibility, and possible diffusion of AuNPs inside the membrane.

## I. INTRODUCTION

In biomedical applications, nanotechnologies provide unique capabilities for drug and gene delivery, diagnosis and treatment of various diseases, biosensors, imaging, and disease therapy. The most clinically established system at the nanometer scale is liposomes, which are single or multi layered lipid bilayer compartments. Since liposomes are biocompatible, biodegradable, along with the capacity for surface and size modification, they are considered an almost ideal drug-carrier system. More importantly, the system can contain both hydrophilic and hydrophobic drugs for delivery, while encapsulation of chemical agents can also reduce toxicity. These systems have been intensively studied due to these potential biomedical applications, and many review articles appear in the literature.^1–3^ A new generation of liposome applications, external or environmental stimuli responsive liposomes, has been designed and prepared for targeted drug release with various imaging capabilities.^4–6^ For such applications, organic, inorganic or metal nanoparticles (NPs) are encapsulated into liposomes to probe new functionality potential.

Among other nanoparticle technologies,^7–10^ gold nanoparticles (AuNPs) are particularly interesting due to their size, shape-, and geometry-controlled synthesis, along with their receptiveness to surface modification. Furthermore, they have distinct electronic properties with tunable optical and x-ray absorption.^11,12^ These properties have various applications in catalysis,^13^ optoelectronics,^14^ biosensors,^15^ drug delivery,^16^ cancer therapy,^17^ imaging,^18,19^ and diagnostics.^20,21^ As the AuNPs can have either a hydrophilically or hydrophobically functionalized surface, these particles can be dissolved in either polar or non-polar conditions. This characteristic allows the nanoparticle, when incorporated into living organisms for example, to have the ability to accumulate in the cytosol or to insert itself into the cell membrane. Understanding the interaction between cell membranes and AuNPs is, thus, an emerging interest.

According to a coarse-grained molecular dynamics simulation, AuNPs with size smaller than a lipid bilayer thickness are captured in the bilayer, while larger NPs with larger lipid-NP interactions, escape from the bilayer.^22^ During this escape, the AuNPs capture some of the lipid molecules from the bilayer, which is a potential reason for cytotoxicity from resulting changes to the membrane composition. The smaller incorporated AuNPs remain in the membrane unless they cluster, whereupon the membrane distortion due to AuNPs in the bilayer is revealed. These modifications to the membrane shape can induce changes in the membrane deformation energy.^23,24^ This consists of two contributions from energy penalties associated with membrane bending and compression. When spontaneously curved bilayers are deformed the bending free energy increases, which is proportional to the bending elastic modulus. Similarly, changes to the lipid bilayer thickness are associated with an increase in the compression free energy, which is proportional to the membrane's compressibility modulus. The insertion of AuNPs in a lipid bilayer is known to change the deformation free energy by modifying the distribution of the alkyl tails of the lipid molecules around the inserted AuNPs, thus, altering these elastic constants.

The membrane's elastic constants dictate the thermal fluctuations of membranes. When AuNPs are attached to giant unilamellar vesicles, Montis *et al.* observed slowdown of the lipid molecular motions which was considered as a rigidification of the lipid bilayers.^25^ More direct observation of membrane dynamics using neutron spin echo (NSE) spectroscopy showed a softening of lipid membranes when silica nanoparticles are attached to the lipid bilayers.^26^ Lipid bilayers with incorporated hydrophobically functionalized AuNPs were studied in terms of the structural and thermal analysis and von White and colleagues suggested a rigidification of the lipid vesicles,^27^ while dynamics measurement by NSE showed softening of the membrane.^28^ The authors for the latter speculated that the membrane softening is induced by increased membrane compressibility resulting from the incorporation of AuNPs in the bilayer. Although it is known that the interactions between membranes and NPs affect not only the elastic properties of the membranes, but also their fluidity,^29^ the influence of NPs on membrane dynamics is not fully understood. It is likely that changes in the conditions, such as charge, particle size, and lipid geometry will affect the dynamical behavior of such membranes.

Lipid bilayers are one of the ideal targets for membrane structure and dynamics studies; however, they form metastable structures, i.e., multilamellar or unilamellar, and the phase behavior of these lipid membranes may be affected by the incorporation of NPs. In order to apply the NSE technique to measure membrane's elastic properties, large unilamellar vesicles are used after undergoing the extrusion procedure.^30^ It has proven difficult to incorporate nanoparticles into membranes due to phase transition of lipid membranes and the aggregation of nanoparticles both inside and outside the membrane.^28^ Furthermore, the weak scattering intensity from unilamellar vesicles has limited detailed studies of the membrane dynamics.

Here, in order to overcome these difficulties, we have used more simplified membranes, composed of a nonionic surfactant, oil, and water. These membranes are thermodynamically stable and the membrane thickness can be easily controlled by varying the amount of oil in the membrane while maintaining the overall lamellar structure.^31–33^ In addition, the scattering intensity is in general strong in the surfactant systems, which allows determination of dynamical parameters with better counting statistics. Hydrophobically modified AuNPs are easily dispersed in the oil phase, so encapsulation in the membrane is straightforward. Yet, at the same time, the surfactant membrane dynamics in this system are fundamentally similar to lipid vesicles.^34^ Thin elastic sheet theories^35–38^ are well applicable to both these surfactant and lipid membranes,^39^ and therefore, the present system well serves as a model membrane for studies into the applications of NP incorporation.

## II. METHODS

### A. Material

Pentaethylene glycol monododecyl ether, C_12_E_5_, with a purity of >98% was purchased from Nikko Chemicals Co., Ltd. In order to control neutron scattering contrast to emphasize scattering signal from different parts of membranes, both protiated and deuterated solvents were used in the present experiments. The *n*-octane-d_18_ (C_8_D_18_), with a purity of 99% and the deuterium oxide, D_2_O, with a purity of 99.9% were purchased from Cambridge Isotope Laboratories, while the n-octane (C_8_H_18_) with a purity of ≥99% was purchased from Sigma Aldrich. The octadecylamine (C_18_H_39_N; ODA) functionalized AuNPs were synthesized in order to facilitate hydrophobic character of the AuNPs. Transmission electron microscopy (TEM) observation showed a mean AuNP diameter of (49 ± 12) Å. A 95% confidence interval of the diameter is from 45.7 Å to 52.3 Å. Thermal gravimetric analysis showed about 20% mass of the AuNPs was from ODA, which allowed us to estimate the grafting density of ODA on 49 Å AuNPs of about 10 molecules/nm^2^.

Dynamic Light Scattering (DLS) measurements were conducted on 0.1% volume fraction solutions of AuNP dissolved in C_8_H_18_ being measured for 10 acquisitions of 50 s at 28 °C. The particle diameter and degree of polydispersity in solution were extracted as 69 Å and 16%, respectively. The estimated hydrodynamic radius from DLS suggests that the ODA extends into the octane phase and the hydrodynamic radius of the AuNP becomes larger than that estimated from the TEM image where the greatest contrast is from the Au core. As seen subsequently, the thickness of the oil-swollen surfactant membrane is roughly the same dimension as the diameter of the AuNPs.

In the present experiments, we have used an oil-swollen lamellar phase to incorporate AuNPs in the membranes as we can easily test the hydrophobic mismatch between the membrane and the AuNP, without changing the size of the particles. The ODA coated AuNPs were successfully dissolved in octane as DLS measurements showed monomodal particle size distribution, and as such, we expect that the nanoparticles are preferentially dispersed in the oil region in the oil-swollen membranes. According to previous experiments on C_12_E_5_/octane/water systems,^33^ the oil-swollen lamellar phase is stable across a wide range of oil content in the membrane when the surfactant volume fraction $\phi_{s}$ is fixed at $\phi_{s}=0.041$ and the amount of oil controls the bilayer thickness. In the present experiments, we changed the bilayer thickness by changing the amount of oil in the membrane to see the effects of membrane-AuNP hydrophobic mismatch on the membrane's structure and dynamics. The values of $\Psi =\phi_{o}/\phi_{s}$ were selected from 0.3 to 1.25, while $\phi_{s}=0.041$ was fixed for the present measurements, where $\phi_{o}$ is the volume fraction of octane. The Au to C_12_E_5_ molar ratios, $X_{Au}=[\text{Au}]/[C_{12}E_{5}$], ranged from 0 to 1 × 10^−3^. For example, the mass composition of each component for Ψ=0.3 and $X_{Au}=1\times 10^{-3}$ was AuNP of 19 *μ*g, C_12_E_5_ of 38 mg, C_8_D_18_ of 9.74 mg, and D_2_O of 1.0475 g. This corresponds roughly to a volume fraction of AuNPs in the solution on the order of $10^{-6}$, while the lamellar membrane is about 2%. Therefore, the scattering intensity is dominated by the lamellar structure and we safely neglect the intensity contribution from the AuNPs in the scattering experiments.

### B. Small-angle neutron scattering

The solution microstructures were quantitatively characterized by small-angle neutron scattering (SANS) measurements which were conducted on the NGB-30m and NG7–30m SANS instruments^40^ at the NIST Center for Neutron Research (NCNR). Data were collected over a momentum transfer, *q*, range between 0.003 Å^−1^ and 0.55 Å^−1^ using neutrons with a wavelength of *λ* = 6 Å and a wavelength distribution Δλ/λ≈0.14, where q=4π sin (θ/2)/λ and *θ* is the scattering angle. The temperature was maintained at a constant 28 °C using a water circulation bath with accuracy greater than 0.1 °C, while the samples were contained within quartz banjo cells with a 1 mm path length. In order to emphasize the membrane structure, C_8_D_18_ was used to form the oil layer so that the neutron scattering contrast of the surfactant–surfactant correlations was enhanced. Maintaining a constant Ψ, a series of samples containing progressively greater mole fractions of AuNP were prepared. The *X_Au_* selected for the SANS measurements was 0, $2.5\times 10^{-5},\;1\times 10^{-4},\;2\times 10^{-4},\;4\times 10^{-4}$, and $1\times 10^{-3}$. The raw two dimensional data were corrected for background scattering, azimuthally averaged, and normalized to an absolute intensity using the SANS data reduction software developed at NIST.^41^ The fit of the experimental SANS data was performed using the SASview software employing the DREAM algorithm to evaluate better parameter correlations and uncertainties.^42^

### C. Neutron spin echo

Neutron spin echo (NSE) spectroscopy offers a unique way of examining membrane motions on the nanometer and nanosecond scales, which is well suited to measure collective membrane fluctuations of these systems. The measurements were conducted on the NGA-NSE spectrometer at the NCNR^43,44^ using *λ* of 6 Å, 8 Å, and 11 Å with a wavelength spread of Δλ/λ≈0.18. The *q* range was covered from 0.04 Å^−1^ to 0.25 Å^−1^, while the measurement time scale *t* was between 0.1 ns and 100 ns. The samples were contained in demountable titanium cells using quartz windows with a 1 mm path length, and the temperature was maintained at 28 °C using an oil circulation system. By selectively deuterating the system, we could focus on different types of collective membrane fluctuations. The C_12_E_5_/C_8_H_18_/D_2_O system provides the scattering contrast of the entire membrane with respect to the solvent D_2_O and is thus sensitive to the membranes' undulation fluctuations in the measured *q* and *t* ranges. On the other hand, the C_12_E_5_/C_8_D_18_/D_2_O system provides additional scattering contrast to see the correlations between surfactant monolayers in an oil-swollen surfactant membrane. Thus, this contrast is sensitive to thickness fluctuations in addition to the undulation fluctuations.^32^ The NSE data were corrected for the polarization of the incoming neutron beam, the resolution function determined by scattering from carbon powder (used as a standard elastic scatterer), and the contribution from the scattering of the bulk D_2_O. This was all done using the Data Analysis and Visualization Environment (DAVE) reduction software.^45^

## III. RESULTS

### A. Structure

#### 1. Effects of oil incorporation

**FIG. 1.**
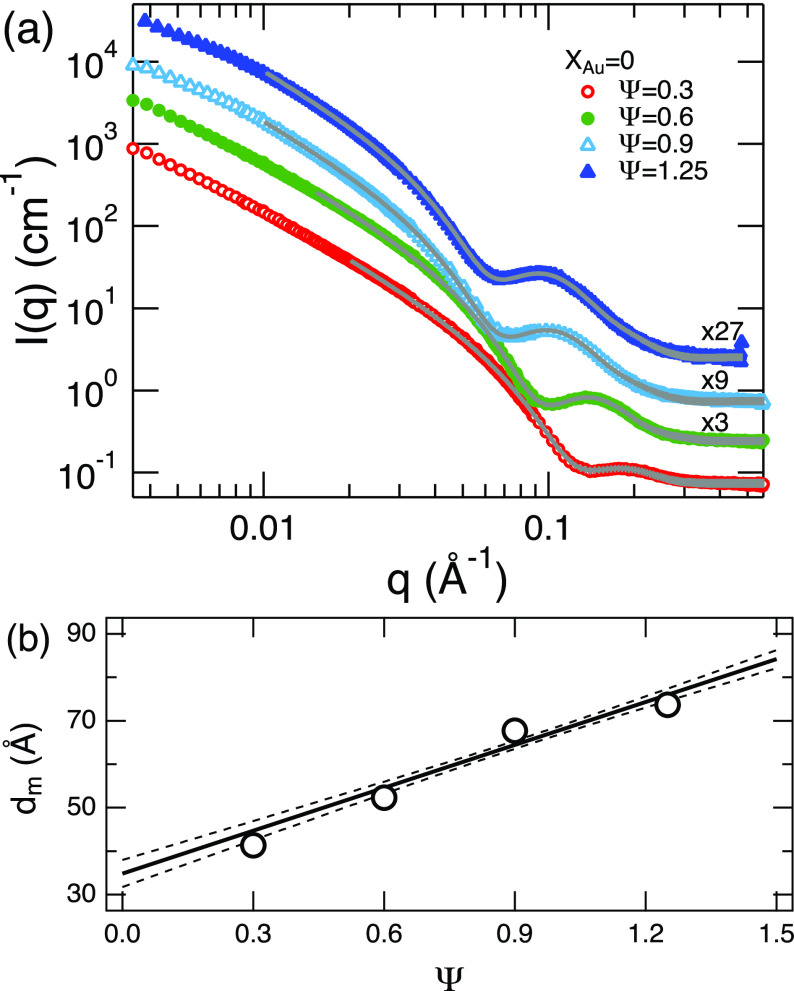
(a) Small-angle neutron scattering profiles for oil-swollen surfactant system forming lamellar structure with varying the amount of oil, Ψ, in the membrane without AuNPs. The solid lines indicate the best fit results to Nallet *et al.* model.^46^ The numbers shown next to the profiles indicate shift factors to the absolute intensity for clarity. (b) The Ψ dependences of *d_m_* are shown. The solid line is a linear fit and the 95% confidence interval is shown as dashed lines. Error bars in this paper represent ±1 standard deviation and are smaller than the symbols in this figure.

Figure 1(a) represents the Ψ dependence of SANS profiles for membranes without incorporated AuNPs. Although the clear elastic scattering peak from the inter-lamellar spacing, which is typically seen in high concentration or well defined lamellar structures, was not observed in the present SANS experiments, the lamellar phase for each sample was confirmed by its optical anisotropy using crossed polarizers prior to the neutron experiments. When Ψ is increased at *X_Au_* = 0, the features seen at high *q* that originate from the form factor of the lamellar membranes shift to lower *q*. This result indicates that the bilayer thickness increases with increasing the amount of oil in the oil-swollen surfactant membrane, which is consistent with the previous results.^33^ The high *q* region was fitted to a form factor model for lamellar bilayers derived by Nallet *et al.*^46^ which permitted quantification of the average bilayer thickness, *d_m_*. This model was developed to fit the scattering intensity for a randomly oriented lyotropic lamellar phase in solution. The solid curves in the figure indicate the respective fit results with fit parameters of the surfactant and oil layer thicknesses, *d_s_* and *d_o_*, the scattering contrast of the oil layer, *ρ_o_*, the oil layer thickness polydispersity, *p_d_*, scale factor, *I_s_*, and the incoherent background level, *I_inc_*.

As Ψ increases, we see an increasing trend of the surfactant layer thickness, *d_s_*. However, the reliability of the estimated single layer thicknesses is less than that of the total bilayer thickness *d_m_*, which is calculated as $d_{m}=2d_{s}+d_{o}$, and shown in Fig. 1(b). The solid line is a linear fit and the dashed lines indicate the range of the 95% confidence interval. The C_12_E_5_ bilayer thickness at Ψ=0 with a 95% confidence interval was estimated to be (34.9 ± 3.1) Å, which is consistent with the previous estimates.^33,47^

#### 2. Effects of AuNP incorporation

**FIG. 2.**
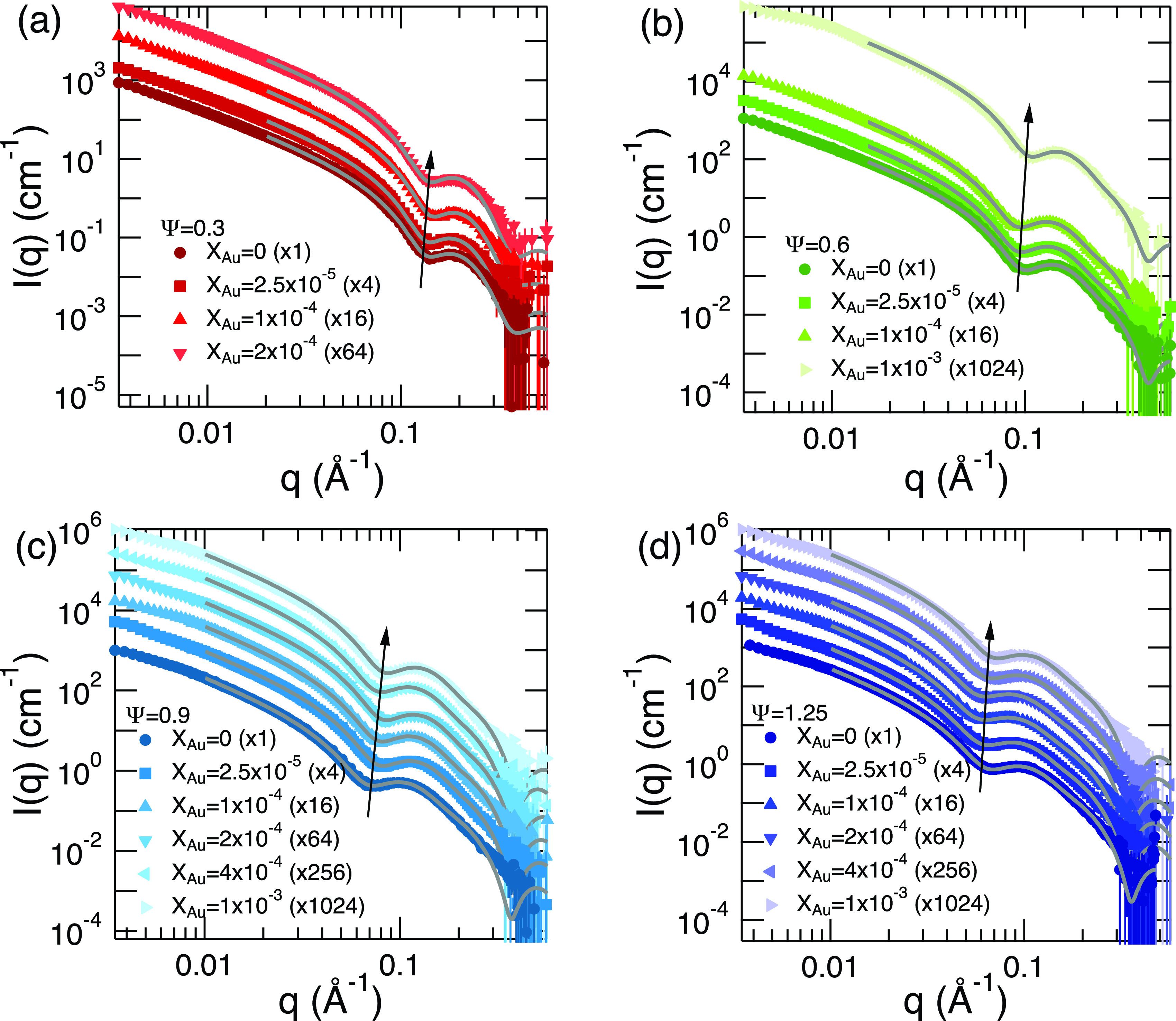
Small-angle neutron scattering profiles for oil-swollen surfactant system forming lamellar structure with incorporated AuNPs in the membrane for both Ψ and *X_Au_* dependencies for (a) Ψ=0.3, (b) Ψ=0.6, (c) Ψ=0.9, and (d) Ψ=1.25, where *I_inc_* was subtracted from the observed scattering intensity. The arrow around the dip location suggests a general shift of the dip location to higher-*q*. Solid lines are the results of the fit to a form factor model lamellar membrane. The parenthesis in the legend shows a shift factor applied to the scattering intensity for clarity. Some error bars are smaller than the symbols.

Figures 2(a)–2(d) show a series of SANS profiles for Ψ=0.3, 0.6, 0.9, and 1.25, respectively, with varying *X_Au_*. The data display similar scattering behavior with increasing AuNP content. A notable feature of the SANS scattering data is the shift to higher *q* values of the dip position that occurs at the length scale corresponding to the average bilayer thickness, *d_m_*. Each plot in Figs. 2(a)–2(d) is also displayed with the corresponding fit to the Nallet model.^46^ The shift of the form factor dip location to higher *q* means the bilayer thickness decreases with increasing AuNPs.

**FIG. 3.**
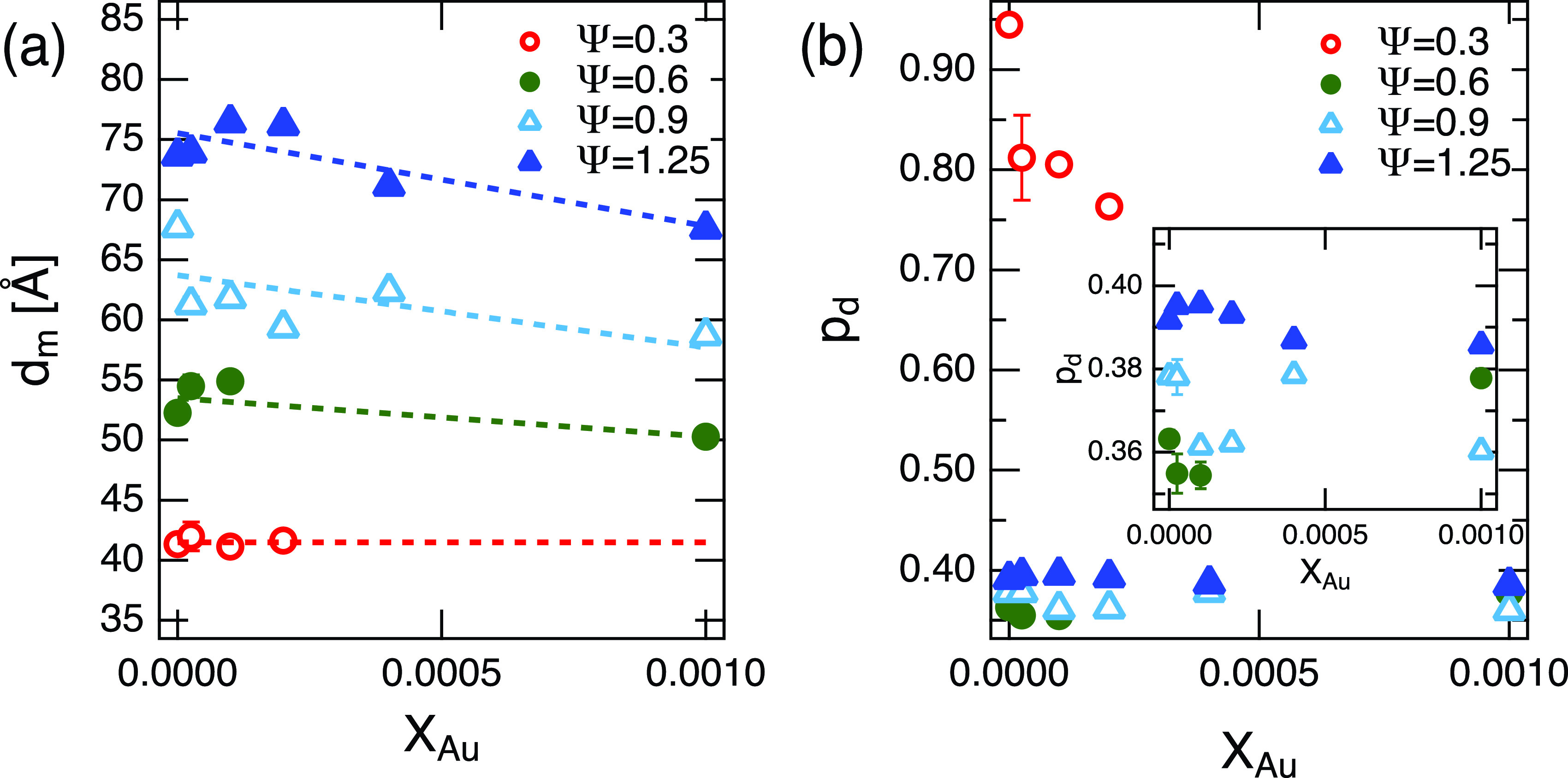
Fit parameters from the SANS measurements. The *X_Au_* dependence of (a) thickness *d_m_* and (b) its polydispersity *p_d_* for the oil-swollen surfactant membrane. The inset to (b) is to show the dependences from Ψ=0.6 to 1.25 samples. Some error bars are smaller than the symbols.

The estimated values of *d_m_* are shown in Fig. 3(a). At small Ψ (Ψ=0.3), where bilayers are less swollen by oil, the value of *d_m_* is almost constant. However, as we increase the amount of oil in the membrane, *d_m_* starts to depend on *X_Au_*. The membrane gets thinner as we increase the amount of AuNP. This contrasts with studies of AuNP loaded lipid bilayers when an increase in the bilayer thickness with increasing AuNP has been observed.^27,28^ Chakraborty *et al.* also pointed that the lipid bilayer thickness changes depend on the particle size, thickening when the particle size is relatively large with no significant change in the thickness for smaller particles.^28^ As we observed thinning effects of AuNPs, the existence of oil in the membrane clearly affects the membrane structure with incorporated AuNPs.

Furthermore, we have estimated the oil layer thickness polydispersity *p_d_* by fitting the data assuming a Gaussian distribution of the bilayer thickness as shown in Fig. 3(b). The values of *p_d_* for Ψ=0.3 samples are significantly bigger than the values estimated for the other samples. This suggests that the sample at Ψ=0.3 has a significantly large heterogeneity in terms of the oil distribution. In addition, the scattering contrast of the deuterated oil region is not significant enough, and estimation of the oil layer and its polydispersity could be more difficult compared to the others. The inset to Fig. 3(b) presents a close-up of the *X_Au_* dependence of the *p_d_* for Ψ=0.6–1.25 samples. In general, the polydispersity becomes slightly smaller if not constant as we add more AuNPs in the membrane. We expect the results of the *X_Au_* dependencies of *d_m_* and *p_d_* to relate to the distribution of AuNPs in the membrane, which will be discussed later in this paper.

### B. Dynamics

#### 1. Undulation fluctuations

**FIG. 4.**
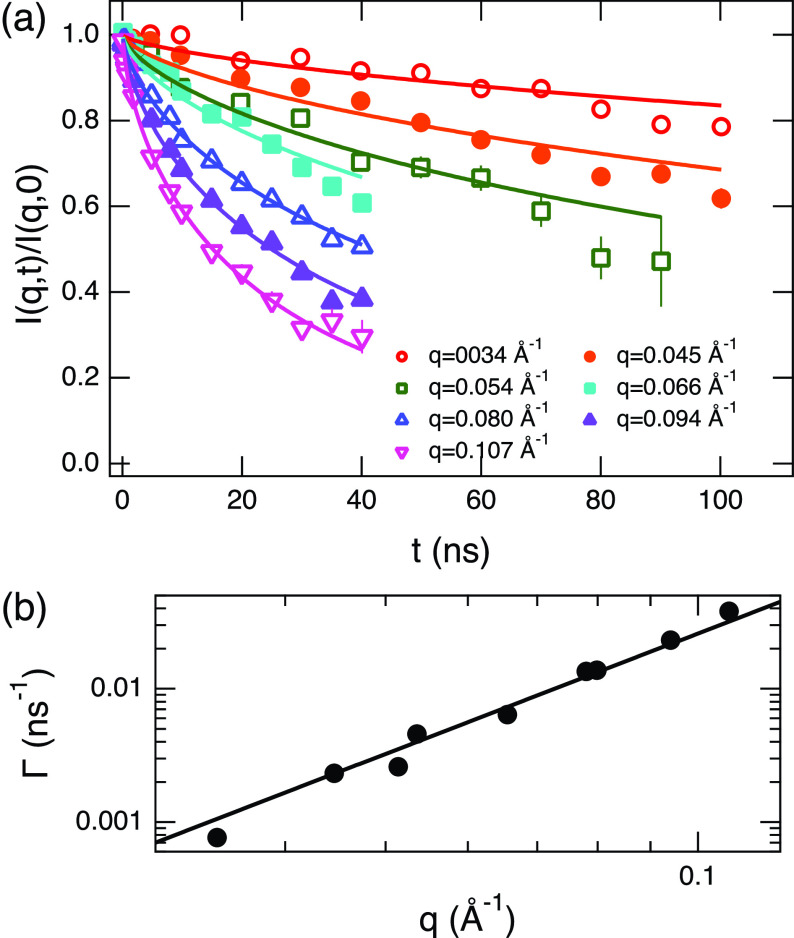
(a) Typical example of the intermediate scattering function, I(q,t)/I(q,0), measured by NSE for C_12_E_5_/C_8_H_18_/D_2_O membrane to measure bending fluctuations. The sample is for Ψ=0.3 and *X_Au_* = 0. The solid lines are the fit results to the Zilman and Granek theory [Eq. (1)]. (b) The observed relaxation rate, Γ, for the sample. The solid line indicates the fit to a power law with the power of 3. Some error bars are smaller than the symbols.

Figure 4(a) displays typical results of the NSE experiments, which measure the normalized intermediate scattering function I(q,t)/I(q,0) of the system for C_12_E_5_/C_8_H_18_/D_2_O. The solid lines in the figure show fit results according to a single membrane fluctuation model proposed by Zilman and Granek (ZG),^48,49^

$$\frac{I(q,t)}{I(q,0)}=\text{exp}\;[-{\left(\Gamma_{ZG}t\right)}^{2/3}],$$ (1)

where Γ_*ZG*_ is the relaxation rate for the single membrane undulation. ZG theory predicts that a non-interacting thin sheet depicts a relationship between the relaxation rate of the bending fluctuations, Γ_*ZG*_, and the bending modulus, *κ*, as,

$$\Gamma_{ZG}=0.025\gamma_{\kappa }\sqrt{\frac{k_{B}T}{\kappa }}\frac{k_{B}T}{\eta_{\mathrm{eff}}}q^{3},$$ (2)

where *η_eff_* is the effective solvent viscosity and $\gamma_{\kappa }$ accounts for the orientational averaging between the membrane plaquettes and scattered neutrons. When $\kappa /k_{B}T\gg 1,\;\gamma_{\kappa }$ approaches unity. However, when $\kappa \approx k_{B}T$, it has been suggested that the full form of $\gamma_{\kappa }=1-3\;\;\text{ln}\;(q\xi_{c})k_{B}T/4\pi \kappa$, where *ξ_c_* is a correlation length, be used.^48^ Furthermore, Monkenbusch and colleagues proposed to analyze NSE data from surfactant membranes to solve numerically the original ZG model so that the estimation of the values of *κ* is more reliable.^50–52^ In the present treatment, though, we set $\gamma_{\kappa }=1$ and apply an effective solvent viscosity to be three times the D_2_O viscosity^53,54^ in order to simplify the discussion. Also, this treatment is the same as our previous study on a similar lamellar system.^31–33^ Although the absolute values of *κ* may have some uncertainties, the relative change between different samples is not affected by such different treatments.

The fit results according to Eq. (1) are shown in Fig. 4(a) by solid lines and the fits are, in general, great. This quality of fit was obtained for all the samples measured. Furthermore, we confirmed that the data for all samples follow the ZG prediction [Eq. (2)] for Γ although the *q*-range required careful consideration. This is because, as we increase Ψ, the bilayer gets thicker and other internal modes come into play at high *q* regions^32^ that are close to the membrane thickness lengthscale. Our estimation of the bending modulus was performed at sufficiently low *q*, where the $\Gamma_{ZG}\propto q^{3}$ relation is satisfied.

**FIG. 5.**
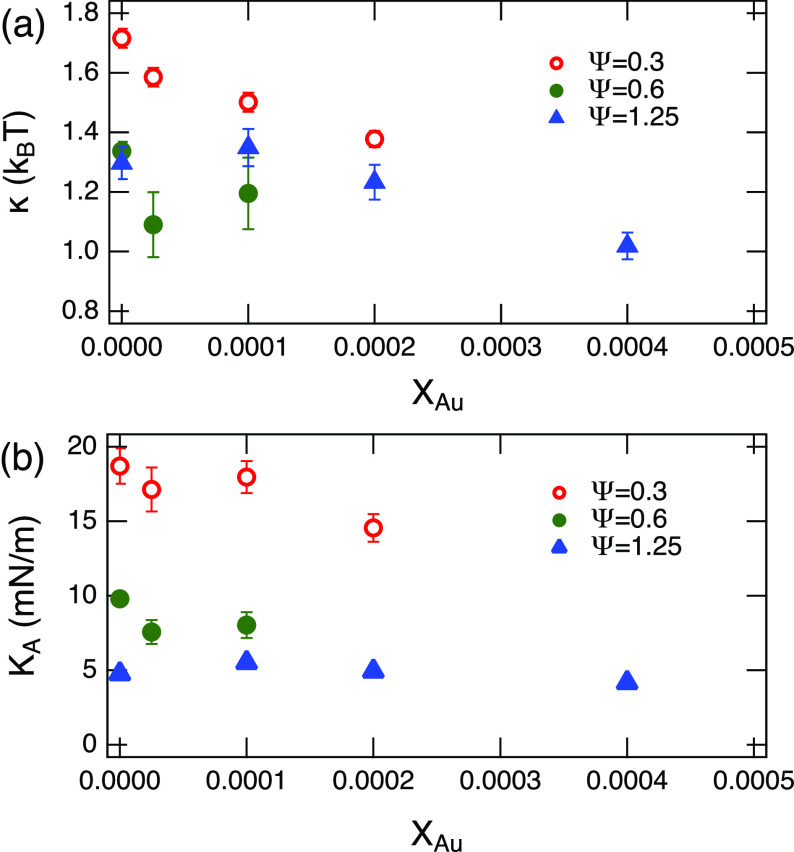
(a) Observed bending modulus *κ* and (b) area compressibility modulus, $K_{A_{\mathrm{bend}}}$ calculated from *κ*. Some error bars are smaller than the symbols.

Values for *κ* calculated from Eq. (2) are displayed in Fig. 5(a). The magnitude of *κ* is close to $k_{B}T$, which is as expected for a surfactant system.^33,55^ When the amount of oil in the membrane increases (increase in Ψ), the bending modulus gets smaller. This behavior is also consistent with the previous observation in a similar system where a maximum bending modulus at Ψ=0.3 was observed.^33^ At low Ψ, the bilayer thickness increases without significantly losing the monolayer coupling, leading to an increase in *κ*. As Ψ increases, the bilayer starts to lose such strong coupling between the monolayers. Eventually, as the proportion of oil in the membrane increases even further, the coupling becomes so weakened that they resemble independent monolayer fluctuations.^33^

There is a notable decrease in the magnitude of *κ* as *X_Au_* increases. Similar softening of the lipid membrane by incorporation of AuNP was reported previously^28^ where the authors observed membrane thickening after incorporation of AuNPs into the bilayers. In thin sheet theory, the membrane stiffness is related to the bilayer thickness in quadratic form, which comes from the idea that bending a membrane induces stretching in the outer leaflet while compressing the inner one.^56^ This general behavior is mathematically expressed as follows:

$$\kappa =\beta d_{m}^{2}K_{A_{\mathrm{und}}},$$ (3)

where *β* and $K_{A_{\mathrm{und}}}$ indicate a coupling constant between leaflets and the area compressibility modulus calculated from the undulation fluctuations, respectively. Here we note $K_{A_{\mathrm{und}}}$ in order to distinguish the values estimated from thickness fluctuations below. The coupling constant *β* is known to span from 1/12 for a fully coupled bilayer (like a slab) to 1/48 for a completely uncoupled bilayer. In the present oil-swollen membranes, we have shown in the SANS results that the oil molecules are condensed in the middle of the bilayer and each surfactant monolayer is already completely uncoupled, thus we assume β=1/48. This assumption is supported by the uncoupling of lipid bilayers by incorporating alkane into a lipid bilayer.^57^

Using Eq. (3), we calculated the values of $K_{A_{\mathrm{und}}}$ as shown in Fig. 5(b). The value of $K_{A_{\mathrm{und}}}$ for Ψ=0.3 series is highest at ≈20 mN/m, which is about an order of magnitude smaller than estimations for lipid bilayers.^58^ As we increase the amount of oil, $K_{A_{\mathrm{und}}}$ becomes even smaller, approaching $K_{A_{\mathrm{und}}}\approx 5$ mN/m for Ψ=1.25. The factor 4× changes of $K_{A_{\mathrm{und}}}$ from Ψ=1.25 to Ψ=0.3 imply that $K_{A_{\mathrm{und}}}$ must be changing even if a potential change in the coupling constant *β* is considered. Polymer brush theory suggests β=1/24 for lipid bilayers.^58^ Therefore, we expect the change in *β* is, at most, a factor 2. This result indicates the change in the bending modulus measured by NSE does not solely come from the change in the membrane thickness and/or inter-leaflet coupling but that the compressibility of membrane is modified with the amount of oil. As we increase *X_Au_*, $K_{A_{\mathrm{und}}}$ slightly decreases, which suggests that the membranes become more compressible with AuNPs. Charkraborty *et al.* proposed an increase in the lipid bilayer compressibility from their results,^28^ and the present results further confirm this speculation. This point becomes even clearer in Sec. III B 2 by measuring thickness fluctuations of the oil-swollen surfactant membranes.

#### 2. Thickness fluctuations

**FIG. 6.**
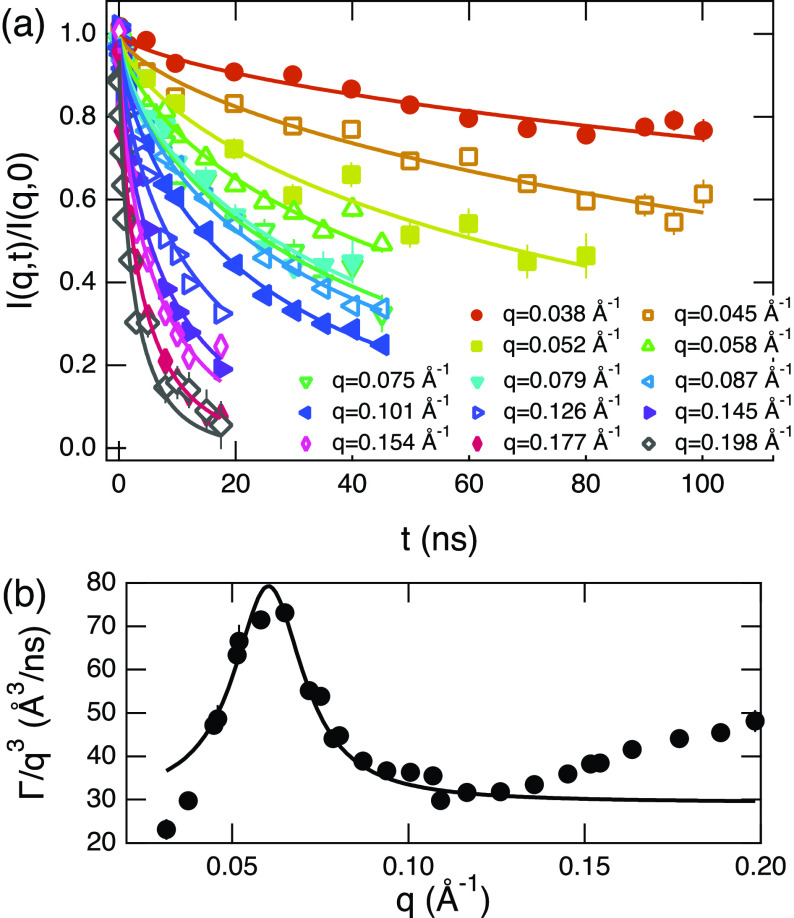
(a) I(q,t)/I(q,0) for C_12_E_5_/C_8_D_18_/D_2_O membrane to measure thickness fluctuations. The sample is for Ψ=1.25 and $X_{Au}=1\times 10^{-4}$. (b) The observed relaxation rate Γ for the same sample which is divided by *q*^3^ to enhance the features of the thickness fluctuations. Some error bars are smaller than the symbols.

We have suggested in Sec. III B 1 that the increase in bilayer flexibility (reduction in *κ*) with Ψ is due to a greater membrane compressibility. An asymmetric membrane theory^38^ predicts that membrane compressibility drives membrane thickness fluctuations, which are measurable by the NSE technique.^31–34,39,59–63^ Figure 6(a) shows I(q,t)/I(q,0) from a system composed of C_12_E_5_/C_8_D_18_/D_2_O. We used the ZG equation [Eq. (1)] to fit the I(q,t)/I(q,0) as shown by solid lines in the figure, and the relaxation rate Γ was extracted, which are shown in the form of $\Gamma /q^{3}$ in Fig. 6(b). If the dynamics solely originated from the undulation fluctuations as discussed earlier, the $\Gamma /q^{3}$ plot should be a constant. However, we clearly see an enhancement in the $\Gamma /q^{3}$ data. The peak like feature that appears at q≈0.06 Å^−1^ has been attributed as thickness fluctuations and fitted with a Lorentz function^31,33,34,59,60^ as

$$\frac{\Gamma }{q^{3}}=\frac{\Gamma_{ZG}}{q^{3}}+\frac{{\left(\tau_{TF}q_{0}^{3}\right)}^{-1}}{1+{\left(q-q_{0}\right)}^{2}\xi^{-2}},$$ (4)

where *τ_TF_* is the relaxation time of the membrane thickness fluctuations, *q*_0_ denotes the position of the peak maximum for the $\Gamma /q^{3}$ plots, and *ξ* denotes the half width at half maximum of the Lorentz function. The first term of Eq. (4) originates from the bending fluctuations as shown in the previous subsection. As *q*_0_ is determined by our SANS experiments, only two fit parameters Γ_*TF*_ and *ξ* are quantified. It is noted here that the upturn in $\Gamma /q^{3}$ observed at high *q* in Fig. 6(b) is due to other internal membrane dynamics, such as the lateral diffusion of surfactant molecules in the membranes, and thus, is excluded from the fitting procedure.

**FIG. 7.**
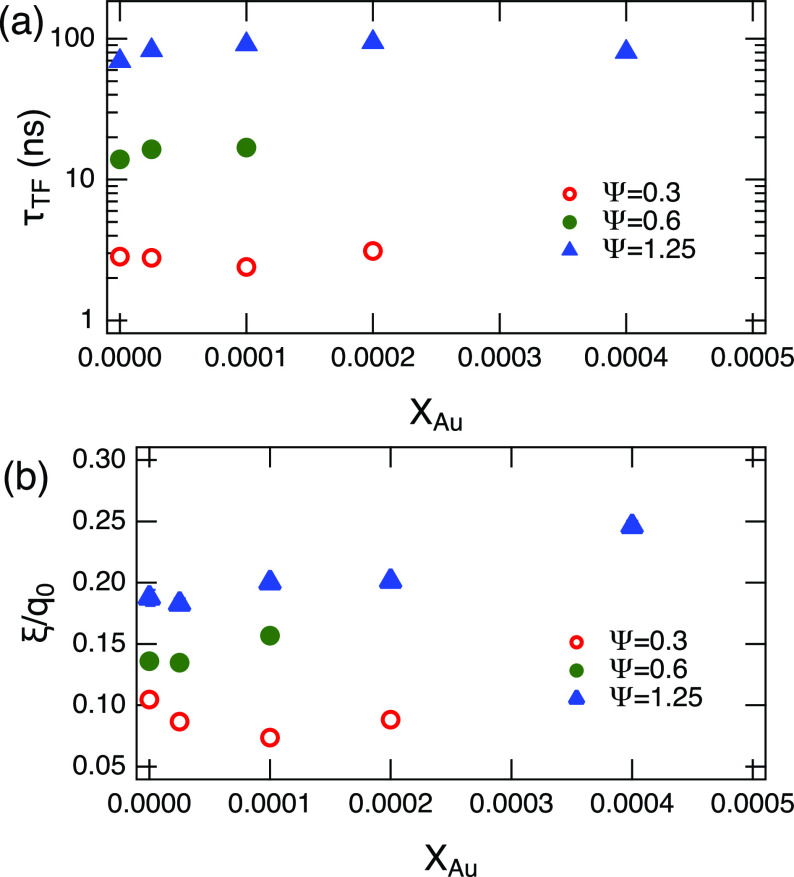
(a) Observed relaxation time of the thickness fluctuations, *τ_TF_*, and (b) the fractional change in thickness $\sigma_{d}=\xi /q_{0}$. Error bars estimated from the fit were smaller than the symbols.

Figure 7 shows the estimated fit parameters from the thickness fluctuations; Fig. 7(a) for *τ_TF_* and Fig. 7(b) for the fractional change in the thickness fluctuation amplitude, $\sigma_{d}=\xi /q_{0}$. When Ψ is increased, both *τ_TF_* and *σ_d_* also increase. As the membrane thickness *d_m_* also increases with the amount of oil, this indicates an increase in the amplitude of the thickness fluctuations with Ψ since the amplitude is expressed as $d_{m}\xi /q_{0}$.^64,65^ Together, these results show that the thickness fluctuations become slower with increasing amplitude as Ψ is increased.

Incorporation of AuNP in the membrane slightly increased *τ_TF_* with *X_Au_*, thus the membrane thickness fluctuations become slower as more AuNP is incorporated in the membrane. On the other hand, an increase in *σ_d_* is clearer for the membranes with Ψ≥0.6, while at low oil content, it decreases with increasing AuNP. This indicates that the presence of the AuNPs with only a small amount of oil has a damping effect on the fluctuations of the membrane, whereas if the amount of oil between the monolayers is sufficiently large, the fluctuation amplitude is enhanced.

**FIG. 8.**
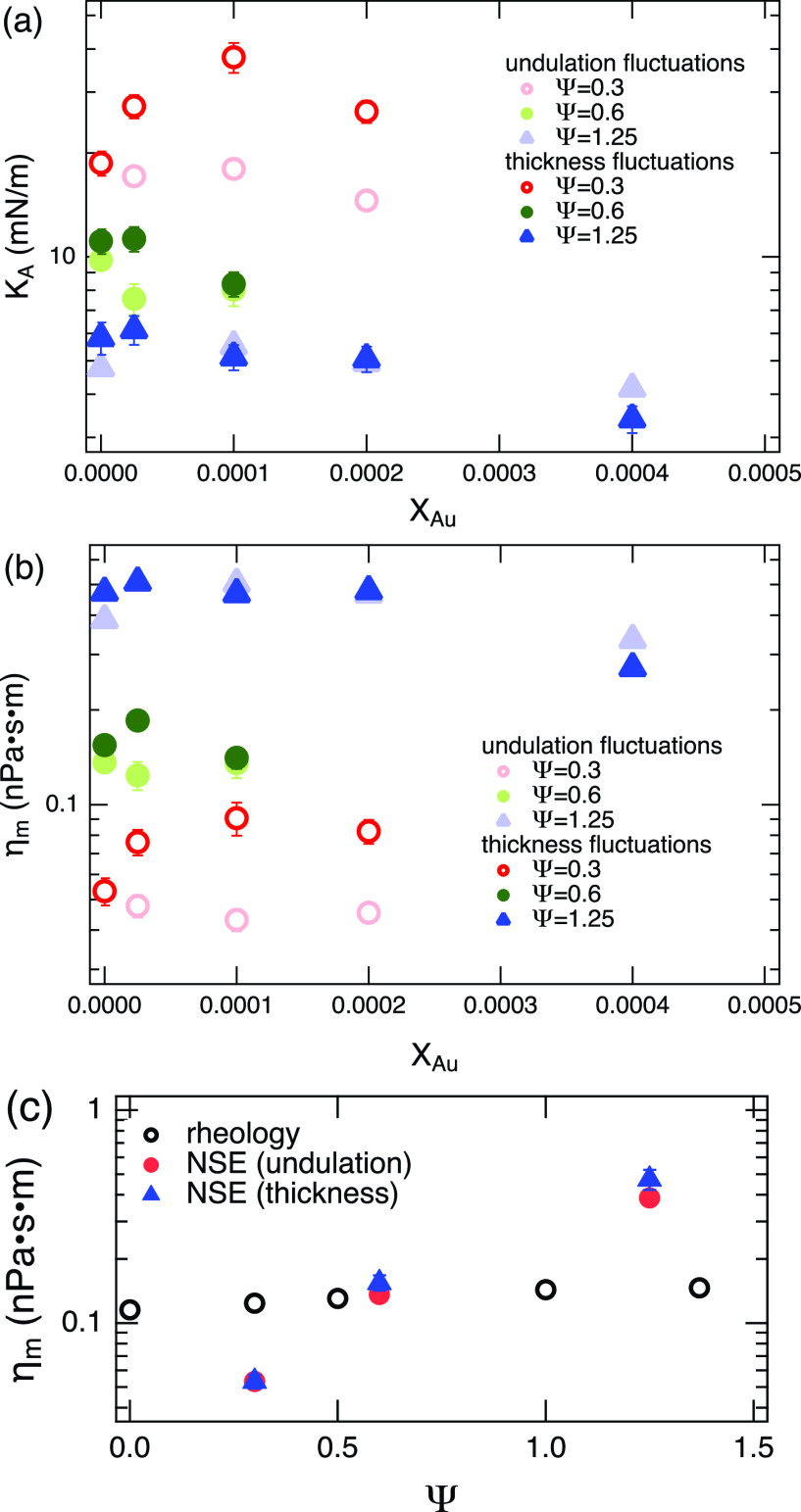
(a) Comparison of area compressibility modulus $K_{A_{\mathrm{und}}}$ and $K_{A_{\mathrm{thick}}}$ and (b) the membrane viscosity *η_m_*. (c) Ψ dependence of the membrane viscosity measured by rheology ($\eta_{m_{b}}$) and NSE (*η_m_*). Some error bars are smaller than the symbols.

Statistical mechanics predicts that the area compressibility modulus relates to the fractional change in area *σ_A_*. If the volume conservation law applies, the fractional change in area is compensated for by the fractional change in thickness. This assumption works well for lipid bilayers both in the presence and absence of oil.^57,66^ Then, $\sigma_{d}=\sigma_{A}$, and the area compressibility modulus can be estimated as

$$K_{A_{\mathrm{thick}}}=\frac{k_{B}T}{\sigma_{d}^{2}A_{L}},$$ (5)

where *A_L_* is the area per molecule. The NSE data include the contribution from the effects of finite instrumental *q* resolution, which is not trivial to take out in the present case, so we normalize the value of $K_{A_{\mathrm{thick}}}$ to $K_{A_{\mathrm{und}}}$ estimated for Ψ=0.3 and *X_Au_* = 0, and the relative change is compared, as shown in Fig. 8(a). With the exception of $\Psi =0.3,\;K_{A_{\mathrm{und}}}$ and $K_{A_{\mathrm{thick}}}$ show similar trends in terms of both Ψ and *X_Au_*. As Ψ increases, *K_A_* decreases significantly. On the other hand, *K_A_* slightly decreased with *X_Au_* except for Ψ=0.3, where $K_{A_{\mathrm{thick}}}$ is increased and it is not consistent with the trends observed for $K_{A_{\mathrm{und}}}$. These results suggest that the incorporation of AuNPs in the oil-swollen surfactant membrane induces increased compressibility of the membrane at a relatively large Ψ.

Bingham *et al.*^38^ predicted that the relaxation time of thickness fluctuations, *τ_TF_*, depends on both *K_A_* and the membrane and solvent viscosities, *η_m_* and *η*. As was originally discussed, for the fluctuations wavelengths shorter than Saffman–Delbrück length, $l_{SD}=\eta_{m}/\eta$,^67^ the in-plane monolayer viscosity dominates and the damping is independent of wavelength,^38^ and in this case the relation is expressed as $\tau_{TF}\approx \eta_{m}/K_{A}$. Figure 8(b) shows the results of calculation of *η_m_* using either $K_{A_{\mathrm{und}}}$ or $K_{A_{\mathrm{thick}}}$.

The values of *η_m_* for the oil swollen surfactant membrane without AuNPs were also estimated from bulk rheology measurements, where the solution composition was set from Ψ=0 to 1.37. The measured bulk viscosity, *η_s_*, was then multiplied by the bilayer thickness, *d_m_*, to calculate $\eta_{m_{b}}=\eta_{s}d_{m}$. The comparison between the NSE and rheology measurements is shown in Fig. 8(c). The rheologically determined $\eta_{m_{b}}$ shows a slight increase with Ψ around 0.1 nPa · s · m. The order of the values estimated by the NSE experiments is consistent with those estimated from the macroscopic rheology measurements. However, the NSE result clearly shows a much steeper increase in *η_m_* with Ψ. A potential explanation of this discrepancy is given in Sec. IV.

## IV. DISCUSSION

### A. Effects of oil in the membrane

In the present experiments, we observed decreases of the bending modulus *κ* as well as the area compressibility modulus *K_A_* with increasing Ψ. Since the bilayer thickness *d_m_* increases with Ψ, the softening of the membrane thus originates from the decrease in *K_A_*. Recently, Nagle^68^ suggested that the bilayer's area compressibility modulus should be considered like a spring for each monolayer and the bilayer compressibility modulus should be sum of the two as per the usual spring force transformation. If we use this, concept the present result cannot be explained, as the bulk modulus of oil is relatively high and the calculated area compressibility modulus of the oil layer is higher than those of surfactant monolayers. If the compressibility modulus was solely coming from the molecular volume compressibility, the oil-swollen surfactant membrane would be less compressible. However, as we have oil molecules in the membranes, they freely move within the pseudo-bilayer. Therefore, the area compressibility modulus of the oil-swollen surfactant bilayers should be considered together with molecular transport degrees of freedom within the membrane. Membranes are much more deformable in the normal plane without violating volume conservation law when oil molecules are in the membrane, i.e., more compressible membranes than those without oil. This trend is also confirmed by the increased thickness fluctuation amplitude, *σ_d_*, thus leading to similar conclusions from both the undulation and thickness fluctuation data sets.

As the bilayer thickness increases, the thickness fluctuation relaxation time *τ_TF_* gets larger. This indicates that the fluctuations become slower as the amount of oil in the oil-swollen bilayer is increased. This result sounds reasonable as larger objects (thicker membranes) usually move slower. However, this result leads to a contradiction in the estimation of the membrane viscosity *η_m_*. As the bulk rheology measurements did not give a significant oil concentration dependence, we do not expect a significant change in the membrane viscosity with increasing amount of oil in the membrane. The original theory by Bingham *et al.* predicts $\tau_{TF}=(\eta_{m}+2\eta /q_{f})/K_{A}$, where *q_f_* is the fluctuation wavenumber.^38^ In addition to the contribution from the membrane viscosity, the solvent viscosity becomes important when the fluctuation wavelength becomes larger than *l_SD_*. By assuming that the estimate for Ψ=0.3 is not affected by this contribution and the *η_m_* does not change significantly with Ψ, we can calculate the fluctuation wavelength $\lambda_{f}=2\pi /q_{f}$ at both Ψ=0.3 and Ψ=1.25 as $\lambda_{f}\approx 200$ nm and $\lambda_{f}∼1\;\mu$m, respectively. The present result therefore suggests an increase in the fluctuation wavelength with Ψ. Due to the large wavelength fluctuations, the relaxation time of the thickness fluctuations become significantly longer at a large Ψ. The discrepancy between rheology and NSE in estimating the membrane viscosity, thus, may originate from the fact that we did not consider a change of fluctuation wavelength, specifically at large Ψ, which might have led to an overestimation of *η_m_* for the NSE data.

### B. AuNP distribution in the membrane

It has been found for lipid systems^69^ that the method of loading the NPs into the membrane can affect the distribution of the NPs inside the membrane. For the present measurements, the AuNP was dissolved into the oil phase and added to the surfactant before bilayer formation, and therefore, we believe the distribution of the AuNPs in the membrane is homogeneous.

The deformation free energy due to the inclusion of hydrophobic nanoparticles into membranes has been described, where the bending and stretching or compression components were considered.^70^ Such a deformation free energy model depicted that a condensed (aggregated) nanoparticle phase in a membrane has the minimum deformation free energy, while there is an energy barrier to reaching a homogeneously distributed (dispersed) nanoparticle state.^23,69^ When the hydrophobic mismatch between membrane and nanoparticle is large, i.e., membrane is thicker than the incorporated nanoparticle size, the deformation free energy gets smaller.

Rasch *et al.*^69^ found that the free energy change required to insert a hydrophobic sphere into a hydrophobic membrane from water, $\Delta G_{\mathrm{solv}}$, is roughly an order of magnitude greater than the free energy penalty incurred in deforming a lipid bilayer by inserting the hydropobic sphere into its center, $\Delta G_{\mathrm{def}}$. A coarse grained molecular dynamics calculation suggested that hydrophobic NPs which are smaller than lipid bilayer thickness are captured in the membrane, unless the NPs are clustered.^22^ In the lipid case, it has been proposed that the mechanism of incorporation involves the lipid hydrocarbon tail of each monolayer leaflet unzipping in order to accommodate the hydrophobic sphere. This creates an energetically unfavorable void space around the NPs. However, in the present oil swollen systems, the oil can fill the void space around the each embedded particle and reduce the $\Delta G_{\mathrm{def}}$ penalty without increasing the deformation of the bilayer hydrocarbon chain conformations, thus we expect that incorporation of AuNP in the membrane is more preferable.

The present SANS and NSE results support the membrane thinning and the increased compressibility of membranes with *X_Au_*. Therefore, we believe that the observed trends of decreasing *d_m_* and *p_d_* with *X_Au_* suggests that the AuNPs do not form a small number of large clusters but rather are dispersed through the membrane individually, or in small clusters, as *X_Au_* increases. This contrasts to a pure surfactant bilayers such as lipid bilayers, in which it can be supposed that a bilayer would favor the condensed state with the AuNPs clustered together to minimize the energy penalty from the void space.

### C. Modification of membrane dynamics by AuNPs

It has been widely thought that the insertion of a rigid additive into a soft membrane should stiffen the membrane.^71–73^ There have been many attempts to confirm the effect of inclusions on the membrane stiffness. Incorporation of cholesterol in lipid bilayers is a typical system that sees stiffening of the membrane.^62,74–77^ n-Alkane and mixed lipid domains in the gel phase are other examples of membrane stiffening.^78,79^ However, n-alkanes and mixed lipids in the fluid phase show the opposite trend, i.e., softening of the bilayer.^57,63^ Moreover, various proteins and peptides also display softening of membranes.^80–85^ There are a couple of theoretical considerations that explain softening of membranes by incorporation of rigid inclusions. Leibler suggest that the presence of diffusing particles coupled to the local curvature of the membrane can reduce the rigidity.^86^ This may be the case for the present system of AuNPs at low Ψ. Another proposal is that there is a modification of the structural arrangement of lipid molecules around the inclusions.^87^ In a previous NSE experiment on lipid vesicles, it was shown that hydrophobically functionalized AuNPs soften the lipid bilayers,^28^ which may be caused by the modification of structural arrangement of lipid molecules. However, in the present oil-swollen lamellar membranes, this mechanism is not a likely origin of the softening as the present system already disrupted the conformation of the surfactant molecules due to the existence of the oil molecules. A likely mechanism is a change of membrane compressibility due to rearrangement of oil molecules surrounding the incorporated AuNP.

On one hand, the change in the thickness fluctuation amplitude is consistent with the change in the area compressibility modulus of the oil-swollen surfactant membranes, but on the other hand, the change in the relaxation time *τ_TF_* with *X_Au_* needs more detailed consideration. As the fluctuation wavelength is likely increased when the amount of oil in the membrane is increased, the AuNP inclusions can affect the fluctuation wavelength as well. If we assume that the change of *τ_TF_* comes from the change in the fluctuation wavelength without modifying the membrane viscosity, we estimate an approximate 20% reduction in the fluctuation wavelength. For example, for Ψ=1.25 samples, $\lambda_{f}\approx 0.8\;\mu$m is expected at the highest AuNP loading. This suggests that the fluctuation wavelength becomes shorter while thickness fluctuations get slower. The opposite trends from the observation of Ψ dependence suggest that there may be another mechanism for inducing such modifications of the thickness fluctuation dynamics.

Another consideration is the effect of AuNP diffusion in the membrane, which also modifies the membrane dynamics. The AuNPs incorporated into membrane have a spherical shape, and applying the Saffman–Delbrück equation^67^ allows estimation of the diffusion constant of an AuNP particle in the membrane. The diffusion coefficient, *D_Au_*, is given as

$$D_{Au}=\frac{k_{B}T}{4\pi \eta_{m}}\left[\text{ln}\;\left(\frac{\eta_{m}}{r\eta }\right)-0.5772\right],$$ (6)

where *r* is the radius of the cylindrical inclusion in the original model and defined here by the hydrodynamic radius, measured by DLS to be r≈35 Å. At the lowest to the highest loadings for Ψ=1.25, *D_Au_* changed from $2.9\times 10^{-12}$ m^2^/s to $4.3\times 10^{-12}$ m^2^/s. This calculation suggests that the AuNPs in the membrane diffuse more quickly with increasing *X_Au_*. In three dimensional diffusion of colloidal particles, it is known that the collective diffusion constant becomes larger when the inter-particle interaction is repulsive.^88,89^ Therefore, the increased *D_Au_* may be consistent with the structural observation where the AuNP particles are in a more dispersed condition rather than condensed state. This may be linked to the increased compressibility at higher AuNP loading as the inclusion mobility may affect the membrane compressibility modulus as well. Although speculative, an interesting outcome from the present experimental results is a suggestion that the lateral diffusion of particles in membrane may affect the membrane dynamics. This point will be followed up in future studies.

Finally, we comment on the *X_Au_* dependence of the structure and dynamics parameters for Ψ=0.3. In order for ODA to swell in membranes, we anticipate that a minimum number of oil molecules are required to surround the AuNPs, which might have been violated at Ψ=0.3. In such a case, the ODA around AuNPs may be in a shrunken state inside the membrane and the inclusions may behave differently than for the well swollen particles. In addition, the bilayer thickness for this sample is $d_{m}\approx 40$ Å, which is similar to the size of the AuNP measured by TEM. However, the DLS measurement provided a hydrodynamic radius of 35 Å, and therefore, in order to incorporate the AuNP particles in the membrane, we expect a significant deformation of the bilayer. In addition, as we see from the polydispersity data [Fig. 3(b)], the amount of oil in each membrane may have a significant distribution. This may lead to a partitioning of AuNPs in the membrane and affect the estimation of the dynamics parameters, specifically the thickness fluctuation amplitude. We believe that although these membranes do contain AuNPs, the estimated structural and dynamical parameters are the average of all the different environments of the encorporated AuNPs, and so not just the dynamics of a single membrane. Even so, the general trends of membrane softening and increased compressibility with increasing AuNP content are consistent with the thicker membranes with more homogeneously distributed nanoparticles.

## V. CONCLUSIONS

In the present study, we have demonstrated that the incorporation of a AuNP into an oil-swollen surfactant bilayer has a significant effect on the dynamics of the bilayer and that the magnitude of these effects are dependent on the concentration of the added AuNP. An increase in *X_Au_* decreases the rigidity of the bilayer which could be interpreted as increased membrane compressibility. The increase in the thickness fluctuation relaxation time is considered to be a result of the increased thickness fluctuation wavelength when the membrane thickness is increased. On the other hand, the inclusion of AuNPs may reduce the fluctuation wavelength. We have shown that NSE can successfully measure these changes to the bilayer dynamics, as well as suggesting that diffusion of inclusions may affect the membrane dynamics. We hope that the ability to successfully differentiate the change in dynamics with NP content can be used as a basis for further work on more complex NP—bilayer systems.

## Data Availability

The data that support the findings of this study are available from the corresponding author upon reasonable request.

## References

[1] M. L. Immordino , F. Dosio , and L. Cattel , “ Stealth liposomes: Review of the basic science, rationale, and clinical applications, existing and potential,” Int. J. Nanomedicine 1, 297–315 (2006). 17717971 PMC2426795

[2] A. Laouini , C. Jaafar-Maalej , I. Limayem-Blouza , S. Sfar , C. Charcosset , and H. Fessi , “ Preparation, characterization and applications of liposomes: State of the art,” J. Coll. Sci. Biotechnol. 1, 147–168 (2012). 10.1166/jcsb.2012.1020

[3] G. Bozzuto and A. Molinari , “ Liposomes as nanomedical devices,” Int. J. Nanomedicine 10, 975–999 (2015). 10.2147/IJN.S68861 25678787 PMC4324542

[4] W. T. Al-Jamal and K. Kostarelos , “ Liposome-nanoparticle hybrids for multimodal diagnostic and therapeutic applications,” Nanomedicine 2, 85–98 (2007). 10.2217/17435889.2.1.85 17716195

[5] Y. Lee and D. H. Thompson , “ Stimuli-responsive liposomes for drug delivery,” WIREs Nanomed. Nanobiotechnol. 9, e1450 (2017). 10.1002/wnan.1450 PMC5557698 28198148

[6] P. S. Zangabad , S. Mirkiania , S. Shahsavaria , B. Masoudia , M. Masroora , H. Hamed , Z. Jafari , Y. D. Taghipour , H. Hashemi , M. Karimi , and M. R. Hamblin , “ Stimulus-responsive liposomes as smart nanoplatforms for drug delivery applications,” Nanotechnol. Rev. 7, 95–122 (2018). 10.1515/ntrev-2017-0154 29404233 PMC5796673

[7] M. S. Martina , J. P. Fortin , C. Ménager , O. Clément , G. Barratt , C. Grabielle-Madelmont , F. Gazeau , V. Cabuil , and S. Lesieur , “ Generation of superparamagnetic liposomes revealed as highly efficient MRI contrast agents for in vivo imaging,” J. Am. Chem. Soc. 127, 10676–10685 (2005). 10.1021/ja0516460 16045355

[8] G. Gopalakrishnan , C. Danelon , P. Izewska , M. Prummer , P. Y. Bolinger , I. Geissbhler , D. Demurtas , J. Dubochet , and H. Vogel , “ Multifunctional lipid/quantum dot hybrid nanocontainers for controlled targeting of live cells,” Angew. Chem., Int. Ed. 45, 5478–5483 (2006). 10.1002/anie.200600545 16847983

[9] Z. R. Stephen , F. M. Kievit , and M. Zhang , “ Magnetite nanoparticles for medical MR imaging,” Mater. Today 14, 330–338 (2011). 10.1016/S1369-7021(11)70163-8 PMC3290401 22389583

[10] K. Bruun and C. Hille , “ Study on intracellular delivery of liposome encapsulated quantum dots using advanced fluorescence microscopy,” Sci. Rep. 9, 10504 (2019). 10.1038/s41598-019-46732-5 31324829 PMC6642191

[11] I. Fratoddi , “ Hydrophobic and hydrophilic au and ag nanoparticles. breakthroughs and perspectives,” Nanomaterials 8, 11 (2017). 10.3390/nano8010011 PMC5791098 29280980

[12] N. Elahi , M. Kamali , and M. H. Baghersad , “ Recent biomedical applications of gold nanoparticles: A review,” Talanta 184, 537–556 (2018). 10.1016/j.talanta.2018.02.088 29674080

[13] G. C. Bond , “ Hydrogenation by gold catalysts: An unexpected discovery and a current assessment,” Gold Bull. 49, 53–61 (2016). 10.1007/s13404-016-0182-8

[14] J. Liao , S. Blok , S. J. van der Molen , S. Diefenbach , A. W. Holleitner , C. Schönenberger , A. Vladyka , and M. Calame , “ Ordered nanoparticle arrays interconnected by molecular linkers: Electronic and optoelectronic properties,” Chem. Soc. Rev. 44, 999–1014 (2015). 10.1039/C4CS00225C 25367894

[15] H. Aldewachi , T. Chalati , M. N. Woodroofe , N. Bricklebank , B. Sharrackc , and P. Gardiner , “ Gold nanoparticle-based colorimetric biosensors,” Nanoscale 10, 18–33 (2018). 10.1039/C7NR06367A 29211091

[16] R. R. Arvizo , S. Bhattacharyya , R. A. Kudgus , K. Giri , R. Bhattacharyaa , and P. Mukherjee , “ Intrinsic therapeutic applications of noble metal nanoparticles: Past, present, and future,” Chem. Soc. Rev. 41, 2943–2970 (2012). 10.1039/c2cs15355f 22388295 PMC3346960

[17] S. Jain , D. G. Hirst , and J. M. O'sullivan , “ Gold nanoparticles as novel agents for cancer therapy,” Br. J. Radiol. 85, 101–113 (2012). 10.1259/bjr/59448833 22010024 PMC3473940

[18] T. Fujiwara , K. Ritchie , H. Murakoshi , K. Jacobson , and A. Kusumi , “ Phospholipids undergo hop diffusion in compartmentalized cell membrane,” J. Cell Biol. 157, 1071–1081 (2002). 10.1083/jcb.200202050 12058021 PMC2174039

[19] J. F. Hainfeld , H. M. Smilowitz , M. J. O'Connor , F. A. Dilmanian , and D. N. Slatkin , “ Gold nanoparticle imaging and radiotherapy of brain tumors in mice,” Nanomedicine 8, 1601–1609 (2013). 10.2217/nnm.12.165 23265347 PMC3657324

[20] I. H. El-Sayed , X. Huang , and M. A. El-Sayed , “ Surface plasmon resonance scattering and absorption of anti-EGFR antibody conjugated gold nanoparticles in cancer diagnostics: Applications in oral cancer,” Nano Lett. 5, 829–834 (2005). 10.1021/nl050074e 15884879

[21] L. A. Dykman and N. G. Khlebtsov , “ Gold nanoparticles in biology and medicine: Recent advances and prospects,” Acta Nat. 3, 34–55 (2011). 10.32607/20758251-2011-3-2-34-55 PMC3347577 22649683

[22] Y. Guo , E. Terazzi , R. Seemann , J. B. Fleury , and V. A. Baulin , “ Direct proof of spontaneous translocation of lipid-covered hydrophobic nanoparticles through a phospholipid bilayer,” Sci. Adv. 2, e1600261 (2016). 10.1126/sciadv.1600261 27847863 PMC5099980

[23] M. Daniel , J. Reznickova , M. Handl , A. Iglic , and V. Kralj-Iglic , “ Clustering and separationof hydrophobic nanoparticles in lipid bilayer explained by membrane mechanics,” Sci. Rep. 8, 10810 (2018). 10.1038/s41598-018-28965-y 30018296 PMC6050295

[24] M. Mendozza , L. Caselli , A. Salvatore , C. Montis , and D. Berti , “ Nanoparticles and organized lipid assemblies: From interaction to design of hybrid soft devices,” Soft Matter 15, 8951–8970 (2019). 10.1039/C9SM01601E 31680131

[25] C. Montis , D. Maiolo , I. Alessandri , P. Bergese , and D. Berti , “ Interaction of nanoparticles with lipid membranes: A multiscale perspective,” Nanoscale 6, 6452–6457 (2014). 10.1039/C4NR00838C 24807475

[26] I. Hoffman , R. Michel , M. Sharp , O. Holderer , M. S. Appavou , F. Polzer , B. Farago , and M. Gradzielski , “ Softening of phospholipid membranes by the adhesion of silica nanoparticles—as seen by neutron spin-echo (NSE),” Nanoscale 6, 6945–6952 (2014). 10.1039/C4NR00774C 24838980

[27] G. V. White , Y. Chen , J. Roder-Hanna , G. D. Bothun , and C. L. Kitchens , “ Structural and thermal analysis of lipid vesicles encapsulating hydrophobic gold nanoparticles,” ACS Nano 6, 4678–4685 (2012). 10.1021/nn2042016 22632177

[28] S. Chakraborty , A. Abbasi , G. D. Bothun , M. Nagao , and C. L. Kitchens , “ Phospholipid bilayer softening due to hydrophobic gold nanoparticle inclusions,” Langmuir 34, 13416–13425 (2018). 10.1021/acs.langmuir.8b02553 30350687

[29] P. B. Santhosh , A. Velikonja , Š. Perutkova , E. Gongadze , M. Kulkarni , J. Genova , K. Eleršič , A. Iglič , V. Kralj-Iglič , and N. P. Ulrih , “ Influence of nanoparticle–membrane electrostatic interactions on membrane fluidity and bending elasticity,” Chem. Phys. Lipids 178, 52–62 (2014). 10.1016/j.chemphyslip.2013.11.009 24309194

[30] Z. Yi , M. Nagao , and D. P. Bossev , “ Bending elasticity of saturated and monounsaturated phospholipid membranes studied by the neutron spin echo technique,” J. Phys.: Condens. Matter 21, 155104 (2009). 10.1088/0953-8984/21/15/155104 21825357

[31] M. Nagao , “ Observation of local thickness fluctuations in surfactant membranes using neutron spin echo,” Phys. Rev. E 80, 031606 (2009). 10.1103/PhysRevE.80.031606 19905122

[32] M. Nagao , “ Temperature and scattering contrast dependencies of thickness fluctuations in surfactant membranes,” J. Chem. Phys. 135, 074704 (2011). 10.1063/1.3625434 21861581

[33] M. Nagao , S. Chawang , and T. Hawa , “ Interlayer distance dependence of thickness fluctuations in a swollen lamellar phase,” Soft Matter 7, 6598–6605 (2011). 10.1039/c1sm05477e

[34] A. C. Woodka , P. D. Butler , L. Porcar , B. Farago , and M. Nagao , “ Lipid bilayers and membrane dynamics: Insight into thickness fluctuationsa,” Phys. Rev. Lett. 109, 058102 (2012). 10.1103/PhysRevLett.109.058102 23006210

[35] W. Helfrich , “ Elastic properties of lipid bilayers: Theory and possible experiments,” Z. Naturforsch., C 28, 693–703 (1973). 10.1515/znc-1973-11-1209 4273690

[36] U. Seifert and S. A. Langer , “ Viscous modes of fluid bilayer membranes,” Europhys. Lett. 23, 71–76 (1993). 10.1209/0295-5075/23/1/012

[37] U. Seifert and S. A. Langer , “ Hydrodynamics of membranes: The bilayer aspect and adhesion,” Biophys. Chem. 49, 13–22 (1994). 10.1016/0301-4622(93)E0077-I

[38] R. J. Bingham , S. W. Smye , and P. D. Olmsted , “ Dynamics of an asymmetric bilayer lipid membrane in a viscous solvent,” Europhys. Lett. 111, 18004 (2015). 10.1209/0295-5075/111/18004

[39] E. G. Kelley , P. D. Butler , and M. Nagao , “ Collective dynamics in model biological membranes measured by neutron spin echo spectroscopy,” in *Characterization of Biological Membranes - Structure and Dynamics*, edited by Nieh M.-P., Heberle F., and Katsaras J. ( de Gruyter, 2019), Chap. 4, pp. 131–176.

[40] C. J. Glinka , J. G. Barker , B. Hammouda , S. Krueger , J. J. Moyer , and W. J. Orts , “ The 30 m small-angle neutron scattering instruments at the National Institute of Standards and Technology,” J. Appl. Crystallogr. 31, 430–445 (1998). 10.1107/S0021889897017020

[41] S. R. Kline , “ Reduction and analysis of SANS and USANS data using IGOR Pro,” J. Appl. Crystallogr. 39, 895–900 (2006). 10.1107/S0021889806035059

[42] See https://www.sasview.org “ SasView for Small-Angle Scattering Analysis” (last accessed September 26, 2020).

[43] M. Monkenbusch , R. Schatzler , and D. Richter , “ The Jülich neutron spin-echo spectrometer—design and performance,” Nucl. Instrum. Methods Phys. Res., Sect. A 399, 301–323 (1997). 10.1016/S0168-9002(97)00956-X

[44] N. Rosov , S. Rathgeber , and M. Monkenbusch , “ Neutron spin echo spectroscopy at the nist center for neutron research,” ACS Symp. Ser. 739, 103–116 (1999). 10.1021/symposium

[45] R. T. Azuah , L. R. Kneller , Y. Qiu , P. L. W. Tregenna-Piggott , C. M. Brown , J. R. D. Copley , and R. M. Dimeo , “ DAVE: A comprehensive software suite for the reduction, visualization, and analysis of low energy neutron spectroscopic data,” J. Res. Natl. Inst. Stand. Technol. 114, 341–358 (2009). 10.6028/jres.114.025 27504233 PMC4646530

[46] F. Nallet , R. Laversanne , and D. Roux , “ Modelling x-ray or neutron scattering spectra of lyotropic lamellar phases: Interplay between form and structure factors,” J. Phys. 3, 487–502 (1993). 10.1051/jp2:1993146

[47] M. Nagao , H. Seto , D. Ihara , M. Shibayama , and T. Takeda , “ Pressure-induced hexagonal phase in a ternary microemulsion system composed of a nonionic surfactant, water, and oil,” J. Chem. Phys. 123, 054705 (2005). 10.1063/1.1993559 16108683

[48] A. G. Zilman and R. Granek , “ Undulations and dynamic structure factor of membranes,” Phys. Rev. Lett. 77, 4788–4791 (1996). 10.1103/PhysRevLett.77.4788 10062631

[49] A. G. Zilman and R. Granek , “ Membrane dynamics and structure factor,” Chem. Phys. 284, 195–204 (2002). 10.1016/S0301-0104(02)00548-7

[50] M. Mihailescu , M. Monkenbusch , H. Endo , J. Allgaier , G. Gompper , J. Stellbrink , D. Richter , B. Jakobs , T. Sottmann , and B. Farago , “ Dynamics of bicontinuous microemulsion phases with and without amphiphilic block-copolymers,” J. Chem. Phys. 115, 9563–9577 (2001). 10.1063/1.1413509

[51] M. Monkenbusch , O. Holderer , H. Frielinghaus , D. Byelov , J. Allgaier , and D. Richter , “ Bending moduli of microemulsions; comparison of results from small angle neutron scattering and neutron spin-echo spectroscopy,” J. Phys.: Condens. Matter 17, S2903–S2909 (2005). 10.1088/0953-8984/17/31/017

[52] O. Holderer , H. Frielinghaus , D. Byelov , M. Monkenbusch , J. Allgaier , and D. Richter , “ Dynamic properties of microemulsions modified with homopolymers and diblock copolymers: The determination of bending moduli and renormalization effects,” J. Chem. Phys. 122, 094908 (2005). 10.1063/1.1857523 15836182

[53] S. Komura , T. Takeda , Y. Kawabata , S. K. Ghosh , H. Seto , and M. Nagao , “ Dynamical fluctuation of the mesoscopic structure in ternary C_12_E_5_-water-n-octane amphiphilic system,” Phys. Rev. E 63, 041402 (2001). 10.1103/PhysRevE.63.041402 11308838

[54] M. C. Watson and F. L. H. Brown , “ Interpreting membrane scattering experiments at the mesoscale: The contribution of dissipation within the bilayer,” Biophys. J. 98, L9–L11 (2010). 10.1016/j.bpj.2009.11.026 20303849 PMC2849052

[55] O. Holderer , H. Frielinghaus , M. Monkenbusch , M. Klostermann , T. Sottmann , and D. Richter , “ Experimental determination of bending rigidity and saddle splay modulus in bicontinuous microemulsions,” Soft Matter 9, 2308–2313 (2013). 10.1039/c2sm27449c

[56] D. Boal , *Mechanics of the Cell* ( Cambridge University Press, 2002).

[57] H. Usuda , M. Hishida , E. G. Kelley , Y. Yamamura , M. Nagao , and K. Saito , “ Interleaflet coupling of n-alkane incorporated bilayers,” Phys. Chem. Chem. Phys. 22, 5418–5426 (2020). 10.1039/C9CP06059F 31904060 PMC7899153

[58] W. Rawicz , K. C. Olbrich , T. McIntosh , D. Needham , and E. Evans , “ Effect of chain length and unsaturation on elasticity of lipid bilayers,” Biophys. J. 79, 328–339 (2000). 10.1016/S0006-3495(00)76295-3 10866959 PMC1300937

[59] R. Ashkar , M. Nagao , P. D. Butler , A. C. Woodka , M. K. Sen , and T. Koga , “ Tuning membrane thickness fluctuations in model lipid bilayers,” Biophys. J. 109, 106–112 (2015). 10.1016/j.bpj.2015.05.033 26153707 PMC4571027

[60] R. Bradbury and M. Nagao , “ Effect of charge on the mechanical properties of surfactant bilayers,” Soft Matter 12, 9383–9390 (2016). 10.1039/C6SM01686C 27830216

[61] M. Nagao , E. G. Kelley , R. Ashkar , R. Bradbury , and P. D. Butler , “ Probing elastic and viscous properties of phospholipid bilayers using neutron spin echo spectroscopy,” J. Phys. Chem. Lett. 8, 4679–4684 (2017). 10.1021/acs.jpclett.7b01830 28892394

[62] S. Chakraborty , M. Doktorova , T. R. Molugu , F. A. Heberle , H. L. Scott , B. Dzikovski , M. Nagao , L. R. Stingaciu , R. F. Standaert , F. N. Barrera , J. Katsaras , G. Khelashvili , M. F. Brown , and R. Ashkar , “ How cholesterol stiffens unsaturated lipid membranes,” Proc. Natl. Acad. Sci. U. S. A. 117, 21896–21905 (2020). 10.1073/pnas.2004807117 32843347 PMC7486787

[63] E. G. Kelley , P. D. Butler , R. Ashkar , R. Bradbury , and M. Nagao , “ Scaling relationships for the elastic moduli and viscosity of mixed lipid membranes,” Proc. Natl. Acad. Sci. U. S. A. 117, 23365–23373 (2020). 10.1073/pnas.2008789117 32883879 PMC7519290

[64] V. Lee and T. Hawa , “ Investigation of the effect of bilayer membrane structures and fluctuation amplitudes on SANS/SAXS profile for short membrane wavelength,” J. Chem. Phys. 139, 124905 (2013). 10.1063/1.4821816 24089802

[65] J. M. Y. Carrillo , J. Katsaras , B. G. Sumpter , and R. Ashkar , “ A computational approach for modeling neutron scattering data from lipid bilayers,” J. Chem. Theory Comput. 13, 916–925 (2017). 10.1021/acs.jctc.6b00968 28080059

[66] M. M. Terzi , M. Deserno , and J. F. Nagle , “ Mechanical properties of lipid bilayers: A note on the poisson ratio,” Soft Matter 15, 9085–9092 (2019). 10.1039/C9SM01290G 31657434

[67] P. G. Saffman and M. Delbrück , “ Brownian motion in biological membranes,” Proc. Nat. Acad. Sci. U. S. A. 72, 3111–3113 (1975). 10.1073/pnas.72.8.3111 PMC432930 1059096

[68] J. F. Nagle , “ Area compressibility moduli of the monolayer leaflets of asymmetric bilayers from simulations,” Biophys. J. 117, 1051–1056 (2019). 10.1016/j.bpj.2019.08.016 31493860 PMC6818143

[69] M. R. Rasch , E. Rossinyol , J. L. Hueso , B. W. Goodfellow , J. Arbiol , and B. A. Korgel , “ Hydrophobic gold nanoparticle self-assembly with phosphatidylcholine lipid: Membrane-loaded and janus vesicles,” Nano Lett. 10, 3733–3739 (2010). 10.1021/nl102387n 20731366

[70] H. S. Wi , K. Lee , and H. K. Pak , “ Interfacial energy consideration in the organization of a quantum dot-lipid mixed system,” J. Phys.: Condens. Matter 20, 494211 (2008). 10.1088/0953-8984/20/49/494211

[71] V. S. Markin , “ Lateral organization of membranes and cell shapes,” Biophys. J. 36, 1–19 (1981). 10.1016/S0006-3495(81)84713-3 7284547 PMC1327573

[72] W. Helfrich and M. Kozlov , “ Flexibility and roughness of mixed and partially polymerized bilayers in terms of the hat model and local bending frustration,” J. Phys. II 4, 1427–1438 (1994). 10.1051/jp2:1994208

[73] R. R. Netz and P. Pincus , “ Inhomogeneous fluid membranes: Segregation, ordering, and effective rigidity,” Phys. Rev. E 52, 4114–4128 (1995). 10.1103/PhysRevE.52.4114 9963884

[74] P. Méléard , C. Gerbeaud , T. Pott , L. Fernandez-Puente , I. Bivas , M. D. Mitov , J. Dufourcq , and P. Bothorel , “ Bending elasticities of model membranes: Influences of temperature and sterol content,” Biophys. J. 72, 2616–2629 (1997). 10.1016/S0006-3495(97)78905-7 9168037 PMC1184459

[75] J. Pan , T. T. Mills , S. Tristram-Nagle , and J. F. Nagle , “ Cholesterol perturbs lipid bilayers nonuniversally,” Phys. Rev. Lett. 100, 198103 (2008). 10.1103/PhysRevLett.100.198103 18518492 PMC2695669

[76] L. R. Arriaga , I. López-Montero , F. Monroy , G. Orts-Gil , B. Farago , and T. Hellweg , “ Stiffening effect of cholesterol on disordered lipid phases: A combined neutron spin echo + dynamic light scattering analysis of the bending elasticity of large unilamellar vesicles,” Biophys. J. 96, 3629–3637 (2009). 10.1016/j.bpj.2009.01.045 19413968 PMC2711415

[77] R. S. Gracià , N. Bezlyepkina , R. L. Knorr , R. Lipowsky , and R. Dimova , “ Effect of cholesterol on the rigidity of saturated and unsaturated membranes: Fluctuation and electrodeformation analysis of giant vesicles,” Soft Matter 6, 1472–1482 (2010). 10.1039/b920629a

[78] M. Hishida , R. Yanagisawa , H. Usuda , Y. Yamamura , and K. Saito , “ Communication: Rigidification of a lipid bilayer by an incorporated n-alkane,” J. Chem. Phys. 144, 041103 (2016). 10.1063/1.4941059 26827195

[79] E. G. Kelley , P. D. Butler , and M. Nagao , “ Scaling of lipid membrane rigidity with domain area fraction,” Soft Matter 15, 2762–2767 (2019). 10.1039/C8SM02362J 30789180 PMC8220873

[80] G. Pabst , S. Danner , R. Podgornik , and J. Katsaras , “ Entropy-driven softening of fluid lipid bilayers by alamethicin,” Langmuir 23, 11705–11711 (2007). 10.1021/la701586c 17939689

[81] H. Bouvrais , P. Méléard , T. Pott , K. J. Jensen , J. Brask , and J. H. Ipsen , “ Softening of popc membranes by magainin,” Biophys. Chem. 137, 7–12 (2008). 10.1016/j.bpc.2008.06.004 18602207

[82] J. Pan , D. P. Tieleman , J. F. Nagle , N. Kučerka , and S. Tristram-Nagle , “ Alamethicin in lipid bilayers: Combined use of x-ray scattering and md simulations,” Biochim. Biophys. Acta 1788, 1387–1397 (2009). 10.1016/j.bbamem.2009.02.013 19248763 PMC2693350

[83] S. Tristram-Nagle , R. Chan , E. Koojiman , P. Uppamoochikkal , W. Qiang , D. P. Weliky , and J. F. Nagle , “ Hiv fusion peptide penetrates, disorders, and softens t-cell membrane mimics,” J. Mol. Biol. 402, 139–153 (2010). 10.1016/j.jmb.2010.07.026 20655315 PMC2940274

[84] J. H. Lee , S. M. Choi , C. Doe , A. Faraone , P. A. Pincus , and S. R. Kline , “ Thermal fluctuation and elasticity of lipid vesicles interacting with pore-forming peptides,” Phys. Rev. Lett. 105, 038101 (2010). 10.1103/PhysRevLett.105.038101 20867811

[85] T. Pott , C. Gerbeaud , N. Barbier , and P. Méléard , “ Melittin modifies bendign elasticity in an unexpected way,” Chem. Phys. Lipids 185, 99–108 (2015). 10.1016/j.chemphyslip.2014.05.004 24875586

[86] S. Leibler , “ Curvature instability in membranes,” J. Phys. 47, 507–516 (1986). 10.1051/jphys:01986004703050700

[87] H. Agrawal , M. Zelisko , L. Liu , and P. Sharma , “ Rigid proteins and softening of biological membranes - with application to HIV-induced cell membrane softening,” Sci. Rep. 6, 25412 (2016). 10.1038/srep25412 27149877 PMC4858729

[88] J. Appell , G. Porte , and E. Buhler , “ Self-diffusion and collective diffusion of charged colloids studied by dynamic light scattering,” J. Phys. Chem. B 109, 13186–13194 (2005). 10.1021/jp051016k 16852643

[89] P. Hopkins , A. Fortini , A. J. Archer , and M. Schmidt , “ The van hove distribution function for brownian hard spheres: Dynamical test particle theory and computer simulations for bulk dynamics,” J. Chem. Phys 133, 224505 (2010). 10.1063/1.3511719 21171689

